# Comprehensive nuclear proteome of *Arabidopsis* obtained by sequential extraction

**DOI:** 10.1080/19491034.2019.1603093

**Published:** 2019-04-09

**Authors:** Chieko Goto, Shoko Hashizume, Yoichiro Fukao, Ikuko Hara-Nishimura, Kentaro Tamura

**Affiliations:** a Graduate School of Agricultural and Life Sciences, University of Tokyo, Tokyo, Japan; b Department of Botany, Graduate School of Science, Kyoto University, Kyoto, Japan; c Department of Bioinformatics, College of Life Sciences, Ritsumeikan University, Shiga, Japan; d Faculty of Science and Engineering, Konan University, Kobe, Japan; e Department of Environmental and Life Sciences, University of Shizuoka, Shizuoka, Japan

**Keywords:** Arabidopsis thaliana, cultured cells, mass spectrometry, nucleus, nuclear bodies, nuclear envelope, nucleolus, proteome

## Abstract

In eukaryotes, the nucleus plays key roles in fundamental cellular processes, including DNA replication, chromatin maintenance, transcription, and translation. To better understand the functional diversity of nuclei, we developed a method for the comprehensive extraction of the nuclear proteome from *Arabidopsis*. We used a buffer with a high sucrose concentration to purify nuclei and then conducted solubility-based fractionation to increase proteome coverage. We identified 1539 proteins and two novel nuclear envelope (NE) proteins in the nuclear fraction of *Arabidopsis* cultured cells. The localization of 25 proteins was determined by GFP fusion analyses; 23 of these proteins were localized either in the nucleus or the NE-associated endoplasmic reticulum. This result was indicative of the high quality of the proteome. These findings will be useful for clarifying novel nuclear functions in plants.

## Introduction

In plants and other organisms, the functions of the nucleus are crucial for cell proliferation and the regulation of gene expression during development and/or in response to biotic/abiotic stresses. Previous studies on vertebrates, yeasts, and protists have focused on different components of the nuclear proteome, including the whole nucleus [1–4], the nucleolus [5–7], the nuclear matrix [8,9], interchromatin granule clusters[10], and the nuclear envelope (NE) [11,12]. Studies on proteins and protein complexes in the nucleus have described the composition and roles of the nuclear pore complex (NPC) [13,14], the spliceosome complex, [15–18] the centromere complex[19], histones, [20] and intrinsically disordered proteins. [21] Other studies have focused on the nuclear proteome under specific conditions, for example, cell proliferation and differentiation[22], differentiation[23], embryonic development[24], organ development, [25] the DNA damage response[26], apoptosis[27], and viral infection. [28–30]

Several groups have conducted nuclear proteomics analyses for various plants [31–35]. In *Arabidopsis* cultured cells, Calikowski et al. identified 36 proteins from nuclear matrix, demonstrating similoarities in protein composition of the nuclear matrix across kingdoms[36]. Bigeard et al. identified a total of 879 proteins, of which 198 were phosphoproteins, in the chromatin-associated fraction from *Arabidopsis*[37]. To identify the stress-regulated nuclear proteins, it was reported the comparison of nuclear proteomes in response to cold stress [38] or MAMP-trigeered immunity[39]. Whereas there are good methods for the extraction and analysis of proteomes from animals, there is still much room to improve methods to extract complete, high-quality proteomes from plants. The largest plant nuclear proteome to date is that of barley with more than 2,400 proteins (deposited in the database; http://barley.gambrinus.ueb.cas.cz/)[40]. Subsequently, more than 800 proteins was identified in rice [41,42] and *Arabidopsis*[37].

In this study, to improve the quality and quantity of the plant nuclear proteome, we used a buffer with a high concentration of sucrose to purify nuclei and then conducted solubility-based fractionation to increase proteome coverage [43,44]. We identified 1539 different proteins from the nuclei of *Arabidopsis* cultured cells and two novel NE proteins. Our approach couples the isolation of pure organelles with solubility-based fractionation to allow comprehensive and efficient identification of the organelle proteome.

## Materials and methods

### Plant materials

*Arabidopsis thaliana* (Columbia-0) was used as the wild type. *Arabidopsis* seeds were germinated on Murashige and Skoog (MS) medium and grown at 22°C under continuous light (35 μmol m^−2^ s^−1^). *Arabidopsis* cultured cells [45] were subcultured in the MS medium containing 2,4-dichlorophenoxyacetic acid at 23°C with continuous agitation in the dark. Tobacco (BY-2) cultured cells were maintained as described previously[46].

## Nuclear isolation from *Arabidopsis* cultured cells

*Arabidopsis* cultured cells (4–5 day old) from 50–100 ml culture were collected and were suspended in 50 ml Cellulase enzyme solution (1% Cellulase ONOZUKA RS, 0.1% pectolyase Y-23, 0.4 M mannitol, 23.4 mM MES-KOH[pH5.7]). The cells were incubated in the Cellulase enzyme solution with shaking (55–65 rpm) at 28°C in dark for 3–4 hours to obtain protoplasts. The protoplasts were filtrated through a 125 µm nylon filter (NIPPON RIKAGAKU KIKAI) and then centrifuged at 1,000 x*g* for 3 minutes. The pellet of protoplasts was suspended in 15 ml Honda buffer (0.44 M sucrose, 2.5% ficoll, 5.0% dextran40, 25 mM Tris-HCl [pH8.0], 10 mM MgCl_2_, 3 mM CaCl_2_, 0.1% Triton X-100, 2.5 mM DTT, 1/50 ml complete EDTA-free) and homogenized with a glass homogenizer. The homogenate was centrifuged at 1,500 x*g* for 10 minutes at 4°C, and the pellet was homogenized in 15 ml Honda buffer. This step was repeated once. The homogenate was filtrated through 80 µm, 30 µm, and 20 µm nylon filters to remove cell debris. The solution passed through the filters was centrifuged at 1500 x*g* for 10 minutes at 4°C. The pellet was re-suspended in 10 ml 2.3 M sucrose buffer (2.3 M sucrose, 3 mM CaCl_2_, 10 mM MgCl_2_, 25 mM Tris-HCl [pH8.0], 0.1% TritonX-100) and centrifuged at 65,000 x*g* for 45 minutes at 4°C. The pellet was re-suspended in Honda buffer and used as ‘isolated nuclei’. Immunostaining and an immunoblot analysis were performed as described previously (anti-histone 1:500, anti-BiP 1:10,000, anti-Nup43 1:1000, anti-PDI 1:2,000)[47]. The signal intensity of each band was semi-quantified with a Fiji software (http://fiji.sc).

### Solubility-based fractionation of nuclear proteins

The isolated nuclei mentioned above were treated with DNase/RNase, and then suspended in a salt solution (1 M NaCl, 25 mM MES [pH5.6], 5 mM MgCl_2_, 10 mM KCl, 0.35 M sucrose, 30% glycerol), followed by incubation for 10 minutes on ice with sometimes stirring. After incubation, the nuclei were centrifuged at 3500 x *g* for 10 minutes. The supernatant was used as salt fraction. The pellet was suspended in an alkaline solution (0.1 M Na_2_CO_3_, 25 mM MES [pH5.6], 5 mM MgCl_2_, 10 mM KCl, 0.35 M sucrose, 30% glycerol), followed by incubation for 10 minutes on ice with sometimes stirring. After incubation, the suspension was centrifuged at 3500 x *g* for 10 minutes. The supernatant is the Alkaline fraction. The pellet was suspended in Triton X-100 solution (1% Triton X-100, 25 mM MES [pH5.6], 5 mM MgCl_2_, 10 mM KCl, 0.35 M sucrose, 30% glycerol), followed by incubation for 10 minutes on ice with sometimes stirring. After incubation, the suspension was centrifuged at 3500 x *g* for 10 minutes. The supernatant is the Triton fraction. The pellet was suspended in Empigen BB solution (0.3% Empigen BB, 25 mM MES [pH5.6], 5 mM MgCl_2_, 10 mM KCl, 0.35 M sucrose, 30% glycerol), followed by incubation for 10 minutes on ice with sometimes stirring. After incubation, the suspension was centrifuged at 3500 x *g* for 10 minutes. The supernatant is the Empigen fraction. The pellet was suspended in SDS solution (4% SDS, 25 mM MES [pH5.6], 5 mM MgCl_2_, 10 mM KCl, 0.35 M sucrose, 30% glycerol), followed by incubation for 10 minutes at room temperature with sometimes stirring. After incubation, the suspension was centrifuged at 3500 x *g* for 10 minutes. The supernatant is the SDS fraction. The remained pellet, which was not solubilized by the procedure mentioned above, was solubilized in 100% formic acid (the formic acid fraction).

### Proteomics and bioinformatics

The proteins in each fraction were precipitated with cold acetone and subjected into in-solution digestion as described previously to obtain the peptides[48]. The peptides were analyzed by liquid chromatography–tandem mass spectrometry using LTQ-Orbitrap XL (Thermo Scientific) as descrived previously[48]. The Identified protein were analyzed with DAVID (https://david.ncifcrf.gov) to annotate GO terms (cellular components). The domain structures were predicted with ATTED-II (http://atted.jp), COILS (https://embnet.vital-it.ch/software/COILS_form.html), and SMART (http://smart.embl-heidelberg.de).

### Plasmid construction

Plasmid DNA construction was performed as described previously[48]. Briefly, to construct the genes for localization analysis of GFP/YFP-tagged proteins by the transient expression, a cDNA fragment encompassing the entire coding sequence of each gene except for a stop codon was amplified from cDNA from flowers of wild-type plants by PCR and cloned into pENTR1A (Invitrogen), and then fused downstream/upstream of sGFP or eYFP tag and downstream of the constitutive cauliflower mosaic virus promoter 35S in plant transformation vectors (pUGW6 or pUGW5). To construct Pro35S:GFP-At1g07970, Pro35S:At1g07970-GFP, and Pro35S:At3g08870-GFP, a genomic fragment containing the entire coding sequence of At1g07970 or At3g08870 without a stop codon was cloned into pENTR1A (Invitrogen) and then fused downstream/upstream of sGFP tag and downstream of the promoter 35S in a plant transformation vector (pGWB406 or pGWB405). The primers used are shown in Supplemental Table S4.

### Transient and stable expression of fusion proteins

For transient expression, protoplasts of *Arabidopsis* cultured cells were transformed with PEG-mediated method[49]. For stable expression, *Arabidopsis* wild-type plants and tobacco BY-2 cells were transformed as described previously [46,50] with *Agrobacterium tumefaciens* (GV3101).

## Results and discussion

### *Isolation of nuclei from* Arabidopsis *cultured cells*

**Figure 1.**
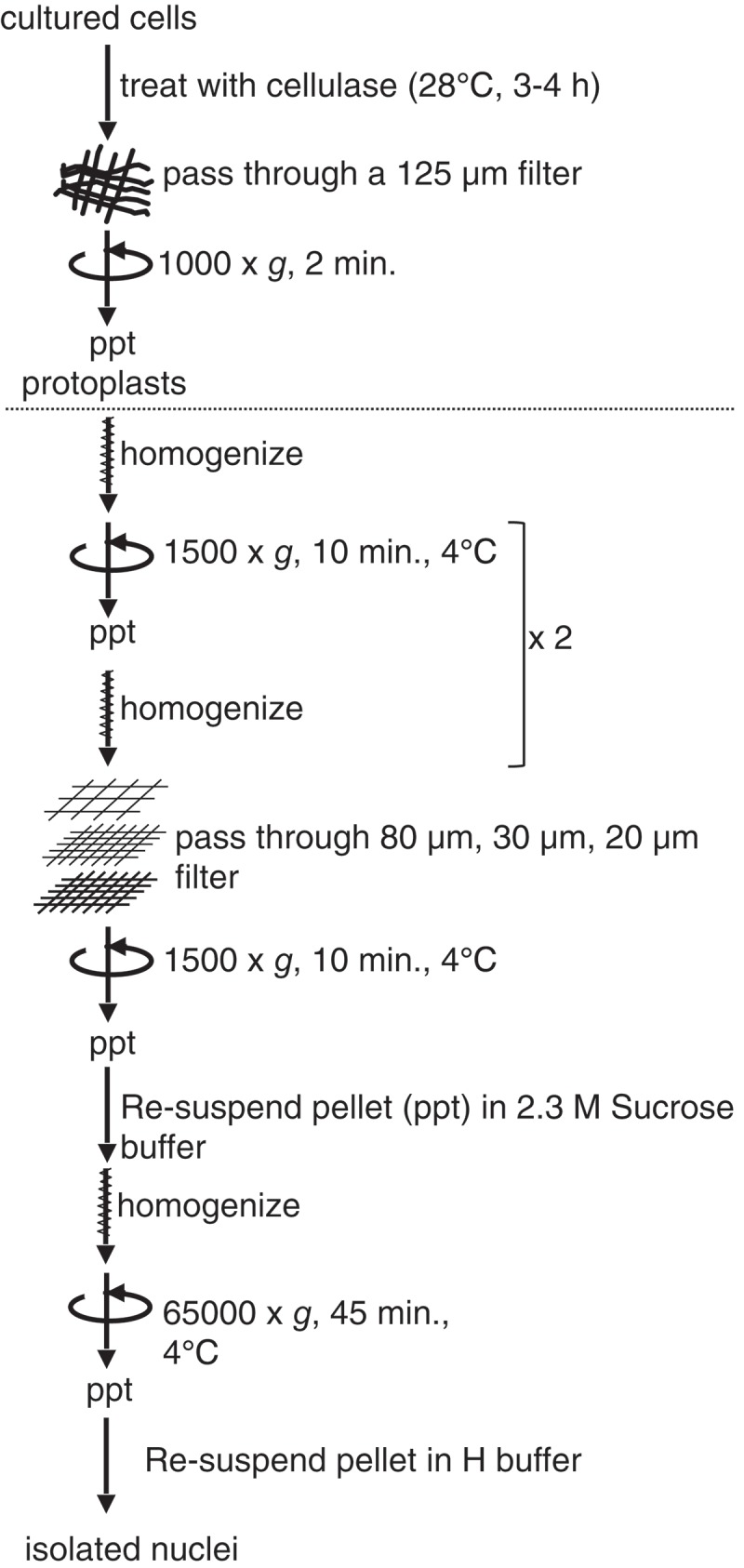
Nuclear isolation procedure used in this study. *Arabidopsis* cultured cells were treated with cellulase solution to obtain protoplasts by degrading cell wall. Protoplasts were passed through a filter, collected by centrifugation, homogenized, and centrifuged again, and then passed through filters. Obtained cell lysate was re-suspended in sucrose buffer, homogenized, and centrifuged to concentrate nuclei. Pellet containing nuclei was re-suspended in Honda (H) buffer. See Materials and methods for details.

**Figure 2.**
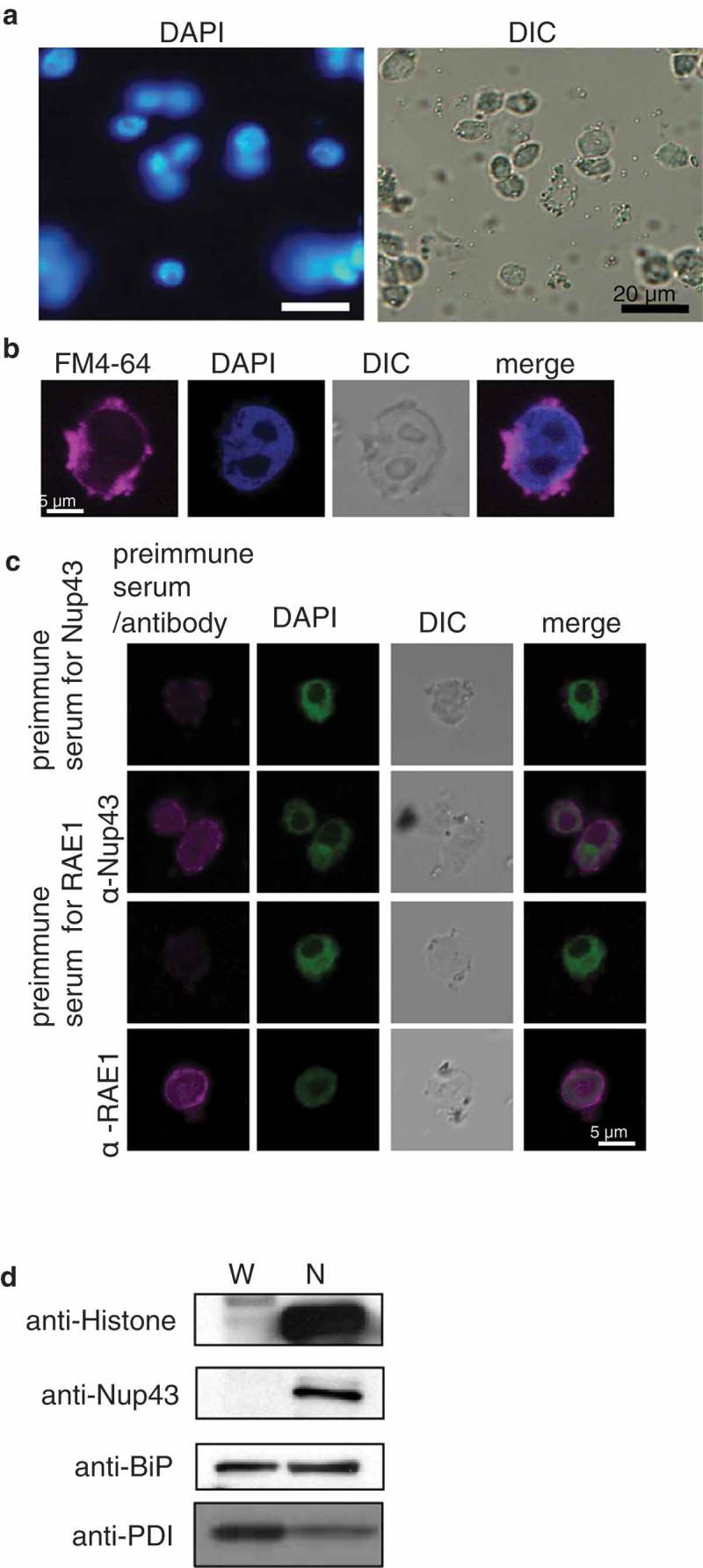
Isolated nuclei. (A) Nuclei isolated by procedure shown in Figure 1 and stained with 4′ 6-diamidino-2-phenylindole (DAPI).(B) Isolated nuclei co-stained with DAPI and FM4-64.(C) Immunostaining of isolated nuclei with preimmune serum (negative control) or antibodies against the nucleoporins Nup43 and RAE1.(D) Immunoblot analysis of whole cell lysate (W) and isolated nuclei (N) with anti-histone antibody (nuclear marker), anti-Nup43 antibody (nuclear envelope marker), anti-BiP antibody (ER luminal marker), and anti-AtALEU antibody (soluble vacuolar marker). DIC, differential interference contrast. Uncropped images of blots are shown in Figure S1.

To isolate sufficient quality of nuclei, *Arabidopsis* cultured cells were used as the starting materials. The nuclear isolation method was based on a solution with a high concentration of sucrose (Figure 1) instead of the Percoll gradient commonly used in previous nuclear proteomics studies [36,38,51]. After isolating cultured protoplasts, a rough nuclear fraction was obtained by a combination of filtration and low-speed centrifugation. Next, we resuspended the fraction in 2.3 M sucrose buffer, and then subjected the mixture to ultracentrifugation to purify the nuclei. The purified nuclei were characterized by fluorescence microscopy after staining with 4′ 6-diamidino-2-phenylindole (DAPI), which indicated successful isolation of nuclei (Figure 2A). Staining with the lipophilic dye FM4-64 confirmed the intact membrane structure of the isolated nuclei (Figure 2B). Immunofluorescent staining detected both Nup43 and RAE1, which are components of the NPC [48,52], in the nuclear rim (Figure 2C). These results confirmed that the isolated nuclei had intact NEs with NPCs.

We then evaluated the purity of the isolated nuclei by immunoblot analyses with antibodies against histone (a nuclear marker), Nup43 (an NE marker), BiP (an ER luminal marker) [53], and AtALEU (a soluble vacuolar marker) [54]. The histone and Nup43 proteins were significantly concentrated in the isolated nuclear fraction (N) compared with the whole cell lysate fraction (W) (Figure 2D and Figure S1). The ER luminal proteins, BiP and PDI, were detected in both fractions, indicative of direct continuity between the lumen of the NE and the ER. The relative signal intensities of BiP and PDI in N to W are 0.77 and 0.57, respectively, suggesting that ER proteins were not concentrated in the nuclear fraction. These results confirmed that nuclei were highly purified by our isolation method.

### Mass spectrometric analyses of nuclear proteome

**Figure 3.**
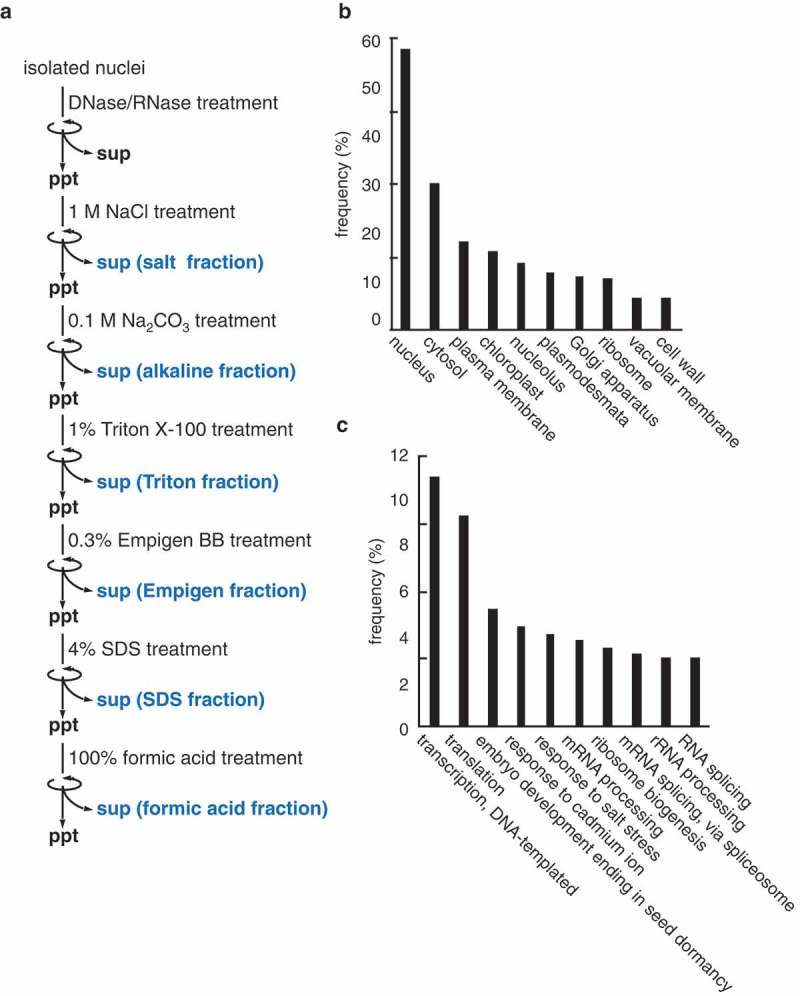
Analysis of nuclear proteome extracted from *Arabidopsis* cultured cells. (a) Solubility-based fractionation of nuclear proteins. Proteins from isolated nuclei were sequentially fractionated, and proteins in each fraction were identified by mass spectrometry.(b) Distribution of 10 most abundant GO terms in cellular component category.(c) Distribution of 10 most abundant GO terms in biological process category.

To increase nuclear proteome coverage, we conducted solubility-based fractionation using sequential extraction with salt, alkaline, detergent, and a strong acid buffer (Figure 3A). The first step was digestion with DNase and RNase to solubilize intranuclear nucleic acids. The salt and alkaline buffers were used to extract the most soluble and peripheral proteins. To extract membrane proteins effectively, we used three types of detergent; Triton X-100, Empigen BB, and sodium dodecyl sulfate (SDS). We attempted to extract insoluble nuclear proteins, which potentially include those required for the formation of functional intranuclear compartments [55], with a formic acid buffer. Each extract was characterized by shotgun proteomics using an LC-MS/MS (LTQ-Orbitrap) system. The mass spectrometry analyses identified 1539 different proteins (Table S1) (189 proteins in the salt fraction, 229 proteins in the alkaline fraction, 1271 proteins in the Triton X-100 fraction, 79 proteins in the Empigen fraction, 345 proteins in the SDS fraction, and 186 proteins in the formic acid fraction) (Figure 3A).

In gene ontology (GO) enrichment analyses, 1450 proteins were annotated in the cellular component category. Among them, 58% were further classified into ‘nucleus’,14.1% into ‘nucleolus’, and 6.6% into ‘ribosomes’ (Figure 3B). This result indicated that nuclear proteins were efficiently concentrated and identified using our preparation and analysis methods. In addition, 162 proteins were annotated in the biological process category (Table S3), suggesting a variety of nuclear functions in plants. Most of them were involved in RNA metabolism (‘transcription’, 10.2%; ‘translation’, 8.6%; ‘mRNA processing’, 3.5%), while others were classified into ‘embryo development (4.7%)’, ‘response to cadmium ion (4.1%)’, and ‘response to salt stress (3.8%)’ (Figure 3C). These results suggested functional differentiation of the plant nucleus between the developmental and stress response pathways.

### Nuclear envelope proteins identified in this study

The NE contains three distinct functional domains; the outer nuclear membrane (ONM), the inner nuclear membrane (INM), and the nuclear lamina. Proteins associated with the NE in plants have been characterized by several groups [56]. *Arabidopsis* has more than 30 nucleoporins, which are components of the NPC associated with the nuclear membrane [48,57]. We identified 14 nucleoporins in our proteomics analysis (Table 1). MAD1, which interacts with the NUA nucleoporin in interphase cells (Ding et al., 2012), was also detected in our proteomics analysis. In addition to nucleoporins, nine known NE proteins were identified (Table 2). The most highly identified NE protein was RANGAP1. The NE localization of RANGAP1 depends on its interaction with the linker of nucleoskeleton and cytoskeleton (LINC) complex composed of WIPs, WITs, and SUNs in *Arabidopsis* [58,59]. In mammalian cells, the LINC complex spans both ONM and INM and associates with the lamina structure [60]. Our proteomics analysis identified four LINC complex constituents (WIT1, WIP3, SUN1, and SUN2) and three putative lamina components (CRWN1, CRWN4, and KAKU4) as NE proteins. These results indicated that our preparation method produced a nuclear proteome with comprehensive coverage of NE proteins.

**Table 1.** nucleoporins identified by MS analysis in this study.

AGI code	Name	Reference
AT5G40480.1	gp210	Tamura et al. (2010)
AT2G41620.1	Nup93a	Tamura et al. (2010)
AT1G79280.1	NUA/TPR	Xu et al. (2007) Plant Cell; Tamura et al. (2010)
AT1G33410.1	Nup160	Muthuswamy and Meier (2011)
AT1G73240.1	NDC1	Boruc et al. (2012)
AT5G51200.1	Nup205	Tamura et al. (2010)
AT1G64350.1	Seh1	Tamura et al. (2010)
AT3G10650.1	Nup136	Tamura et al. (2010)
AT3G14120.1	Nup107	Tamura et al. (2010)
AT4G32910.1	Nup75	Tamura et al. (2010)
AT1G24310.1	Nup54	Tamura et al. (2010)
AT2G30050.1	Sec13	Tamura et al. (2010)
AT4G37130.1	Nup58	Tamura et al. (2010); Ferra´ndez-Ayela et al. (2013)

**Table 2.** NE proteins identified by MS analysis in this study (except for nucleoporins).

AGI code	Name	Reference
AT3G63130.1	RANGAP	Rose and Meier (2001); Xu et al. (2007) Current Biology; Xu et al. (2008); Zhou et al. (2012)
AT1G67230.1	CRWN1	Dittmer et al. (2007); Sakamoto and Takagi (2013) (;Ciska et al. [2013];) Graumann (2014)
AT5G04990.1	SUN1	Graumann et al. (2010); Oda and Fukuda (2011); Graumann (2014); Graumann et al. (2014); Tatout et al. (2014); Zhou et al. (2015)
AT5G65770.1	CRWN4	Sakamoto and Takagi (2013)
AT4G31430.1	KAKU4	Goto et al. (2014)
AT5G11390.1	WIT1	Zhao et al. (2008); Zhou and Meier (2014); Zhou et al. (2015)
AT5G49880.1	MAD1/NES1	Ding et al. (2012)
AT3G10730.1	SUN2	Graumann et al. (2010); Oda and Fukuda (2011); Zhou et al. (2015)
AT3G13360.1	WIP3	Xu et al. (2007) Current Biology

**Figure 4.**
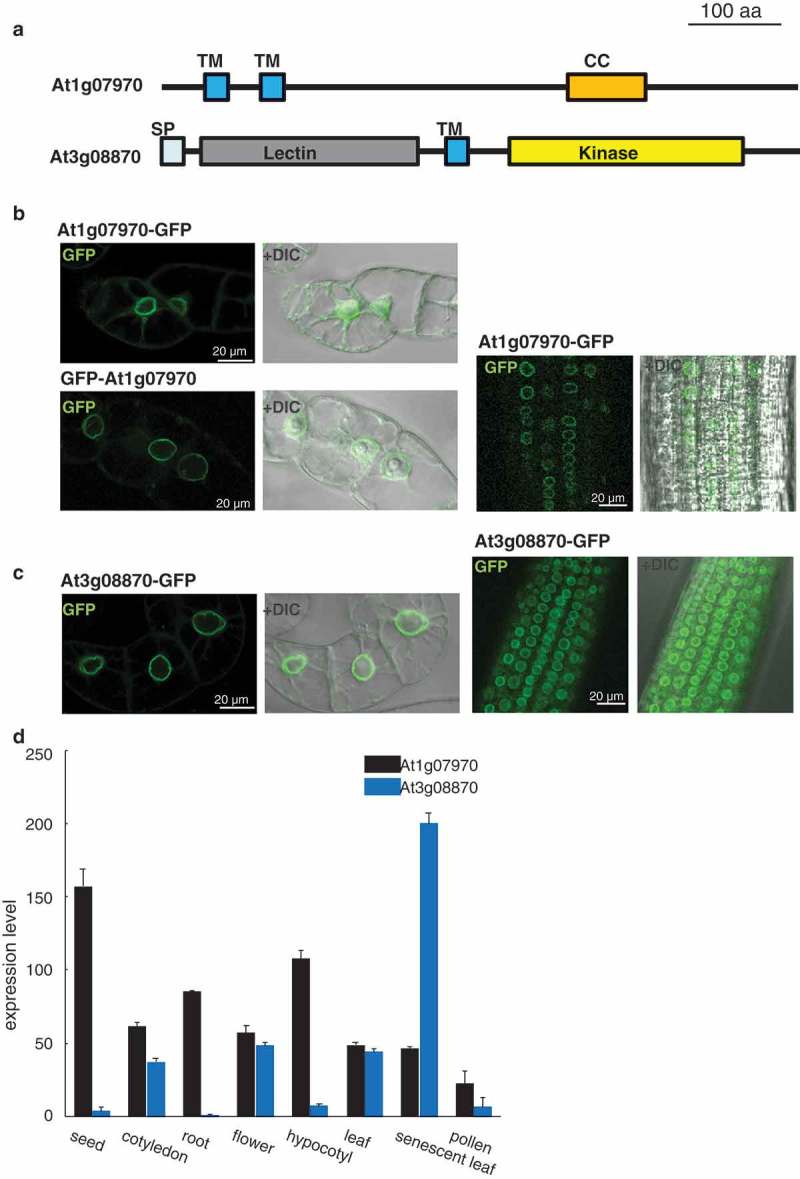
Identification of two novel nuclear envelope proteins. (a) Schematic representation of At1g07970 and At3g08870 proteins. At1g07070 has putative two transmembrane domains (TM) and a coiled-coil domain (CC). At3g08870 protein has putative signal peptide (SP) followed by lectin domain, transmembrane domain (TM), and kinase domain.(B and C) Fluorescence images of tobacco BY-2 cells and *Arabidopsis* root cells stably expressing GFP-tagged At1g07970 (b) or At3g08870 (c). DIC, differential interference contrast.(d) Transcript levels of *At1g07970* and *At3g08870* in various tissues. Data were obtained from resource page of AtGenExpress project (http://jsp.weigelworld.org/AtGenExpress/resources/) [73]. Error bars indicate standard deviation.

In our nuclear proteome, we identified two novel NE proteins, At1g07970 and At3g08870, with putative transmembrane domains (Figure 4A). At1g07970 had two transmembrane domains at the N-terminal and a short coiled-coil region in the center of the protein. At3g08870 had a signal peptide, a lectin domain, a transmembrane domain, and a kinase domain. Stable expression of GFP-fused proteins revealed that they exclusively localized on the NE in tobacco BY-2 and *Arabidopsis* root cells (Figure 4B). Searches of publicly available microarray data revealed that At1g07970 is broadly expressed in vegetative and reproductive tissues while At3g08870 is highly expressed in senescent leaves. This result suggested that At3g08870 is involved in a specific function of the NE during the senescence process. This is the first report of a kinase-domain-containing protein localized on the NE in plants. Based on its primary structure, At3g08879 was predicted to be a type-I integral membrane protein with its kinase domain located at the cytoplasmic side. It would be interesting to explore the role of kinase signaling on the NE in the senescence signaling pathway.

### Localization analyses of proteins with coiled-coil or transmembrane domains

**Figure 5.**
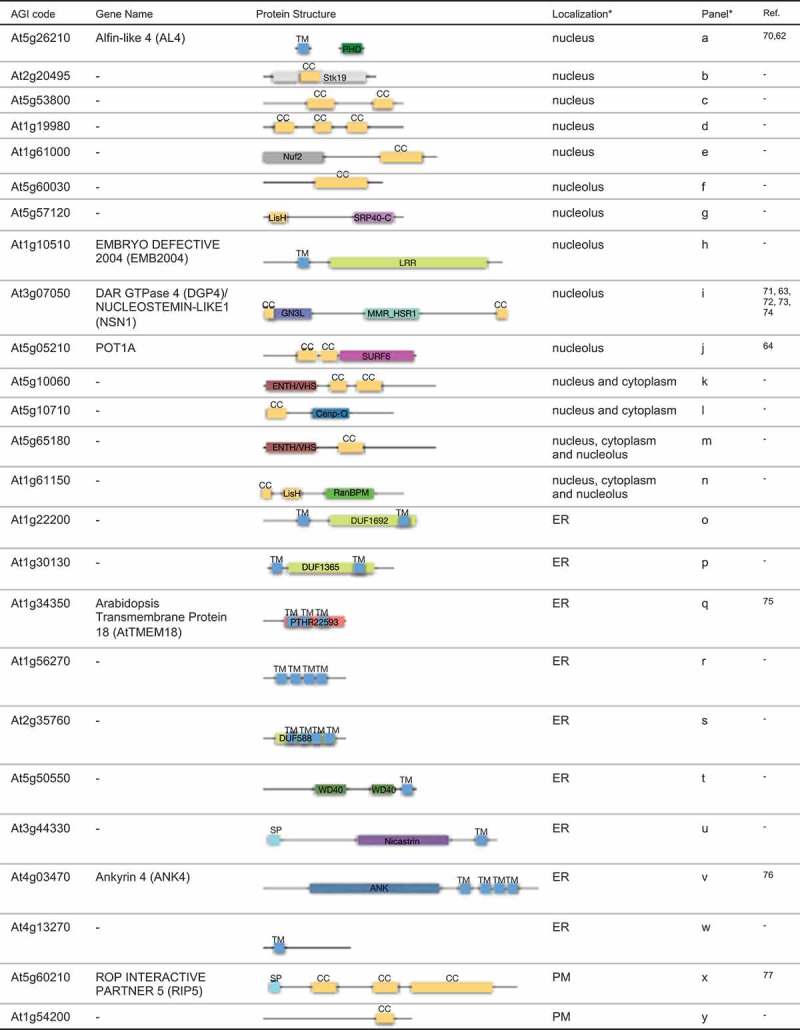
Schematic representation of predicted domain features of proteins characterized in this study. Localization analysis is shown in Figure S2. References are follows: 1[74].; 2[64].; 3[75].; 4[65].; 5[76].; 6[77]., 7[78].; 8[66].; 9[79].; 10[80].; 11[81].

Nuclear coiled-coil proteins are involved in providing the nucleoskeleton and molecular scaffolds that organize membrane systems [61], while integral membrane proteins on the NE play multiple roles in shaping the nuclear membrane [62,63]. To identify novel proteins involved in the functional organization of the nucleus, we selected 25 proteins with either a coiled-coil domain (14 proteins) or a transmembrane domain (11 proteins; Figure 5) and analyzed their subcellular localization by transient expression in *Arabidopsis* cultured cells. Five proteins (At5g26210, At2g20495, At5g53800, At1g19980, and At1g61000) fused with GFP were localized in the nucleus. Among them, At5g26210 (AL4) has been reported to localize to the nucleus previously [64], while the other four proteins (At2g20495, At5g53800, At1g19980, and At1g61000) were newly characterized in this study (Figure S2). Five proteins (At5g60030, At5g57120, At1g10510, At3g07050, and At5g50210) fused with GFP were localized in the nucleolus exclusively. At3g07050 (Nucleostemin-like1: NSN1) has been reported to localize in the nucleolus and regulate the cell cycle [65], while At5g05210 (Protection of telomerase 1: POT1A) has been reported to localize both to the cytoplasm and to the nucleus [66]. POT1A (At5g05210) interacts with the nucleolar protein TERT-V (I8) [66], indicating that POT1A is able to localize the nucleolus under certain conditions. Four proteins (At5g10060, At5g10710, At5g65180, and At1g61150) showed diverse patterns of localization, including in the nucleus, nucleolus, and cytoplasm. It is possible that these proteins are trafficked between the nucleus and cytoplasm. Indeed, both At5g10710 and At5g65180 had an Epsin N-terminal homology (ENTH/VHS) domain, whose structure is similar to those of karyopherin and beta-katenin [67], which are known to be shuttled between the nucleus and the cytoplasm [68]. The GFP fusions of nine proteins with a transmembrane domain showed a typical NE and ER-localization pattern. Two coiled-coil proteins (At5g60210 and At1g54200) fused to GFP localized in dot-like structures at the PM. Consistently, At5g60210 (RIP5) has been identified in another PM proteomics analysis [69]. Together, the results of these GFP-fusion localization analyses revealed that 23 out of 25 analyzed proteins were localized in either the nucleus or NE-associated ER. In the transient expression system, fusion proteins can be highly accumulated in the cell. Therefore, we could not exclude the possibility that the expressed fusion proteins were leaked from nuclear membrane to ER membrane. Stable expression system under control of own promoter will be further required for assessing exact protein localisation.

A successful proteomic analysis of complex biological samples is often hindered by high-abundance proteins[70]. Therefore, the ability to selectively deplete high-abundance proteins for efficient detection of minor proteins is increasingly important in proteomic studies[44]. Our sequential fractionation based on protein solubility is a powerful approach to reduce proteome redundancy after enrichment of not only nucleus but also other organelles. Indeed, for protein preparation in 2 dimensional electrophoresis, sequential extraction strategies are universally exploited, most with alternations in pH and ionic strength of extraction buffers[71]. Recently, Blavet et al. created a barley (*Hordeum vulgare* L., cv. Morex) nuclear protein database [40]. This database contains large numbers of proteins identified from nuclei at the G1, S, and G2 cell cycle phases, and follows on from their previous study reporting 803 proteins [72]. Their database and the results obtained in our study provide comprehensive information about plant nuclear proteins.

## Abbreviations

ER: endoplasmic reticulum
GO: gene ontology
INM: inner nuclear membrane
ONM: outer nuclear membrane
NE: nuclear envelope
NPC: nuclear pore complex(es)
PM: plasma membrane
