# Supplementary figures and images for: Comprehensive nuclear proteome of Arabidopsis obtained by sequential extraction
Source: Nucleus. 2019 Apr 9;10(1):81–92. doi: 10.1080/19491034.2019.1603093 (PMC6527390; doi:10.1080/19491034.2019.1603093)

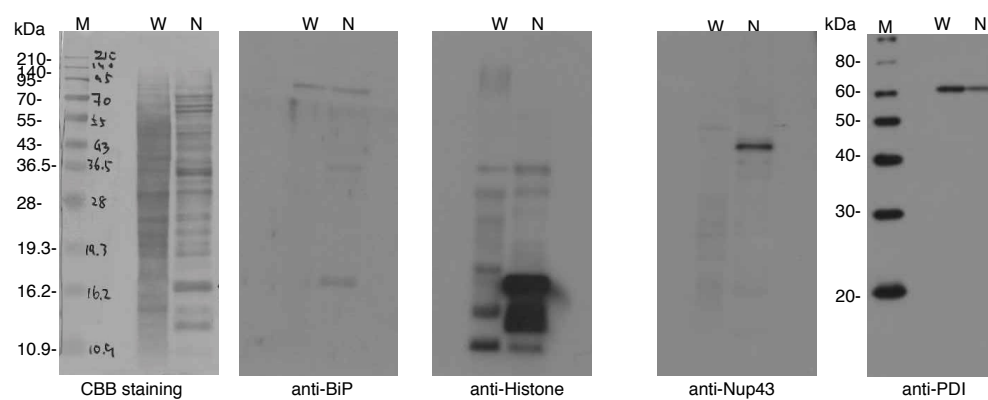

Fig. S1.

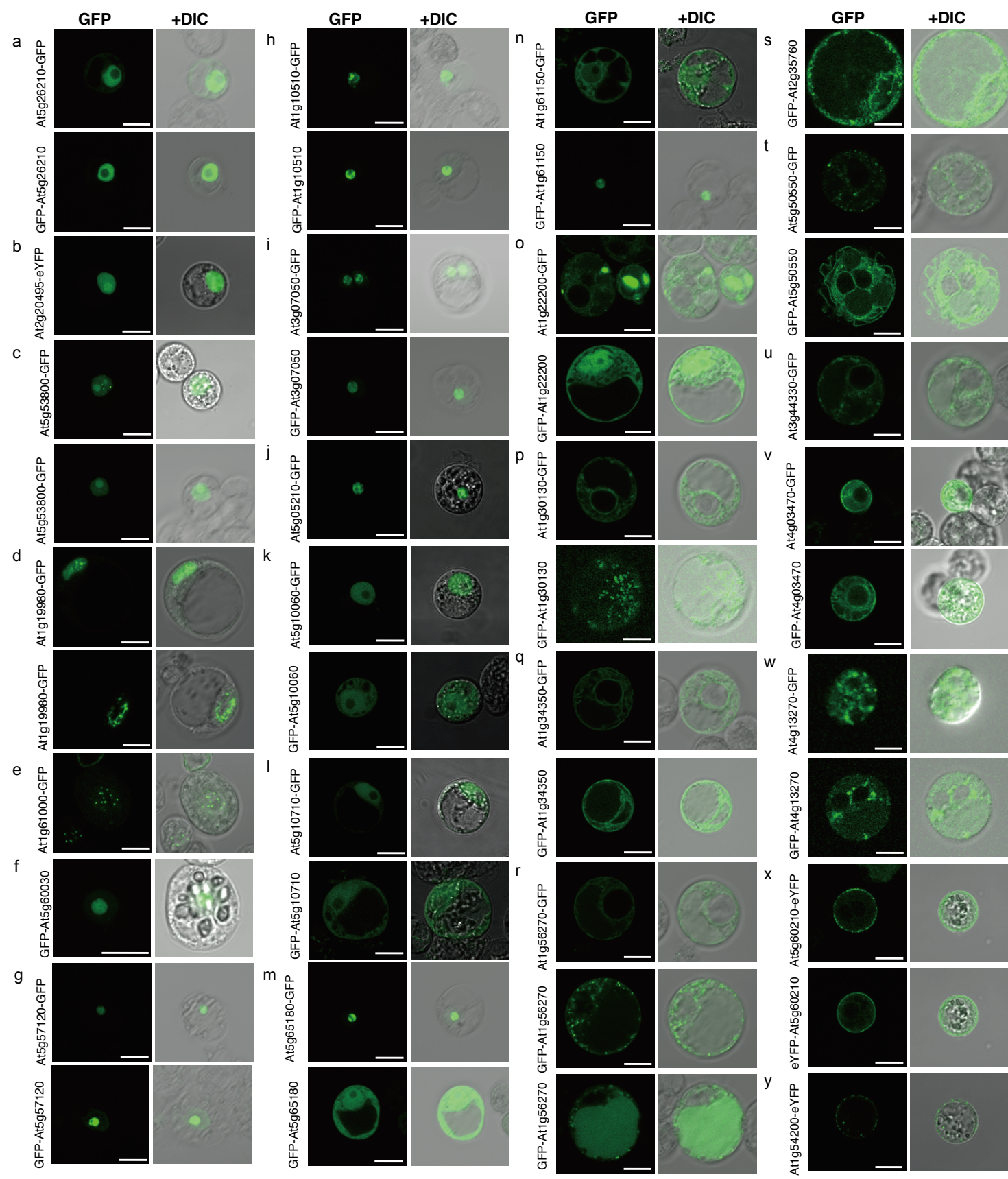

Bars = 10  $\mu$ m

Fig. S2.

Supplement: Supplemental Material [file kncl-10-01-1603093-s001.zip › Supplementary information/190326 Tamura_SupplementalFigures..pdf]
